# A Rare Case of Bladder Metastasis From Endometrial Cancer Treated With Robot-Assisted Radical Cystectomy

**DOI:** 10.7759/cureus.59713

**Published:** 2024-05-06

**Authors:** Naotaka Kumada, Makoto Kawase, Keita Nakane, Tatsuhiko Miyazaki, Takuya Koie

**Affiliations:** 1 Urology, Matsunami General Hospital, Gifu, JPN; 2 Urology, Gifu University Graduate School of Medicine, Gifu, JPN; 3 Pathology, Gifu University Hospital, Gifu, JPN

**Keywords:** immunostaining, complete surgical resection, robot-assisted radical cystectomy, endometrial cancer, metastatic bladder cancer

## Abstract

Malignant tumors metastasizing to the bladder are uncommon, and bladder metastasis from uterine cancer is particularly rare. Several cases of bladder metastasis from malignant melanoma, gastric cancer, breast cancer, and renal cancer have been documented. However, to our knowledge, only four cases of bladder metastasis from endometrial cancer had been reported up until 2024. Here, we present a case of bladder metastasis of endometrial cancer following modified radical hysterectomy, which was successfully treated through multidisciplinary intervention.

## Introduction

Endometrial cancer is prevalent among middle-aged women and is usually diagnosed at an early stage [1]. Many early-stage cases are successfully treated through surgery, and the postoperative recurrence rate is approximately 13% [1]. Notably, most recurrences occur within the first three years of initial treatment, and late-stage recurrences are rare [1]. Endometrial cancer often metastasizes to the vagina, lungs, liver, peritoneum, and lymph nodes but rarely to the bladder [1]; to our knowledge, only four such cases had been reported up until 2024 [2]. Here, we present the case of a 50-year-old woman with endometrial cancer who underwent a robot-assisted radical cystectomy (RARC) a decade after her initial surgery.

## Case presentation

A 50-year-old woman underwent radical hysterectomy and pelvic lymph node dissection in 2011 for stage II uterine cancer. Pathological examination revealed endometrioid adenocarcinoma, Grade 1, pT3N0M0. Postoperative adjuvant therapy was not administered, and the patient had regular check-ups.

Seven years postoperatively, a tumor near the transverse colon was detected by abdominal computed tomography (CT) and was suspected to be due to peritoneal dissemination. Pelvic magnetic resonance imaging (MRI) showed thickening of the left side wall of the bladder. A transurethral resection of the bladder tumor (TURBT) was performed, and histopathological analysis indicated metastatic endometrioid adenocarcinoma with the invasion of the muscular layer of the bladder. The patient was diagnosed with bladder metastasis of endometrioid adenocarcinoma with peritoneal dissemination. Subsequently, she underwent six courses of combination chemotherapy with docetaxel and carboplatin. The peritoneal dissemination temporarily regressed but then gradually returned; thus, the peritoneal tumor was resected laparoscopically for local control. Histopathological analysis confirmed that the dissemination was from the primary endometrioid adenocarcinoma. She was diagnosed as having achieved a complete response (CR) to chemotherapy because the bladder tumor had disappeared on imaging studies.

**Figure 1:**
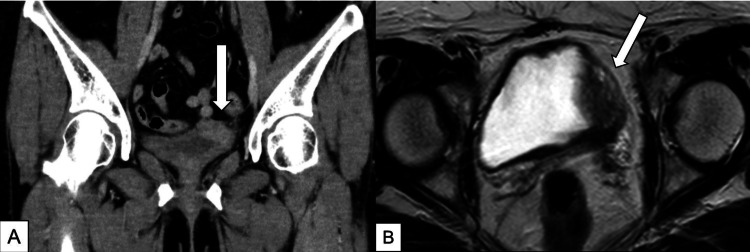
Abdominal CT (A) and pelvic MRI (B) revealed a tumor lesion with partial extravesical infiltration on the left wall of the bladder CT: computed tomography; MRI: magnetic resonance imaging

However, four months after laparoscopic surgery, she experienced gross hematuria and consulted a urologist. Abdominal CT and pelvic MRI revealed a bladder tumor lesion with partial extravesical infiltration of the left bladder wall (Figure 1A, 1B).

**Figure 2:**
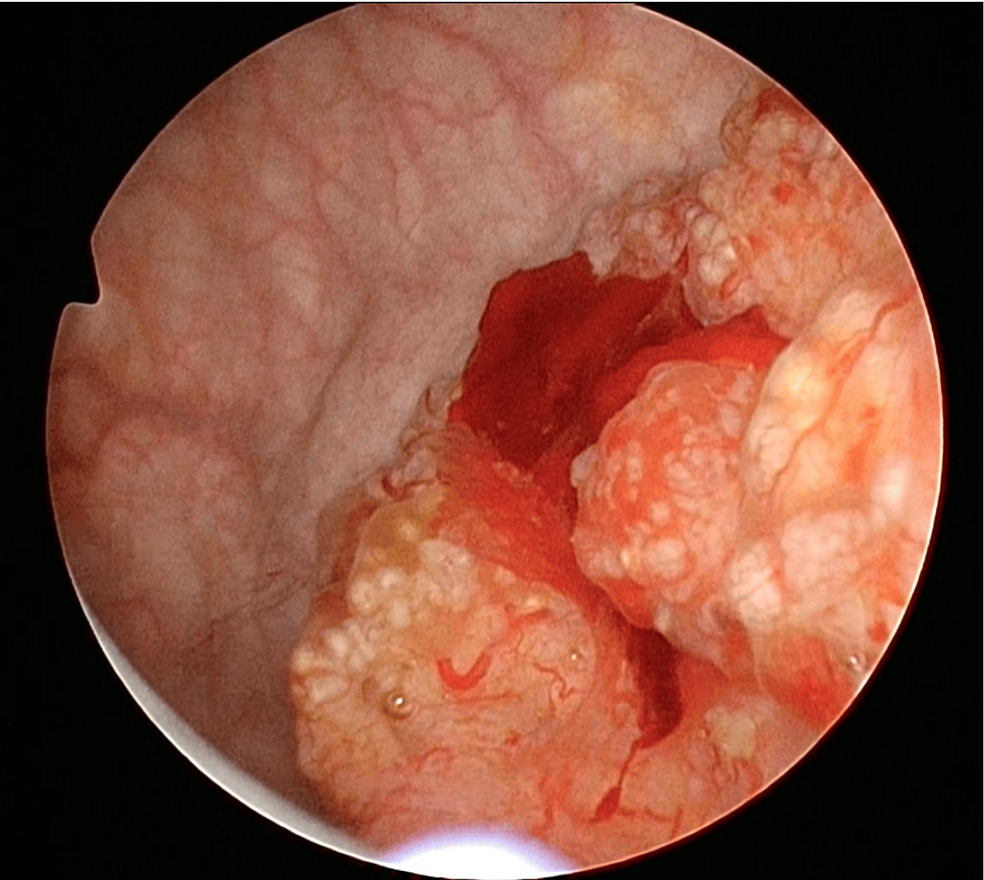
Cystoscopy revealed a non-papillary tumor on the left wall

Urine cytology, hematology, and biochemistry were normal. The CA-125 level was 6.8 U/mL (normal range, <35.0 U/mL), and the CA19-9 level was 5.0 U/mL (normal range, <37.0 U/mL). Cystoscopy revealed a non-papillary tumor on the left bladder wall (Figure 2). 

**Figure 3:**
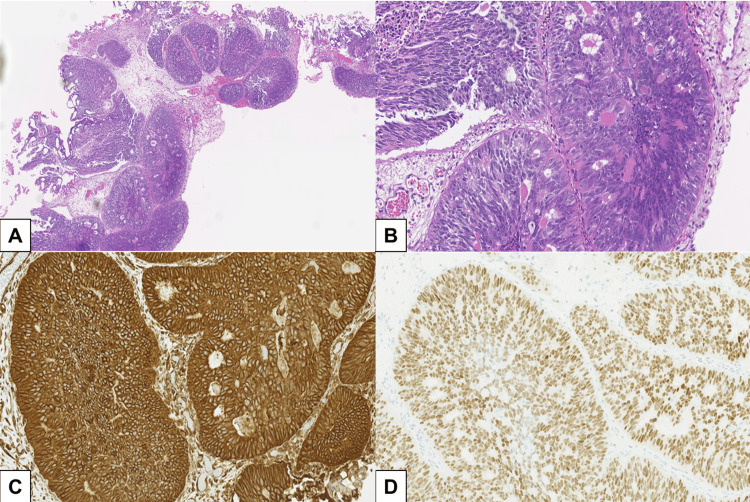
HE staining reveals glandular-tubular structures fused to papillary configurations within the bladder muscle layer (A and B). Immunohistochemical analysis revealed positive staining for CK7, vimentin (C), and estrogen receptor (D) and negative staining for CK20 and p63. The morphological features and immunostaining profiles suggest metastatic endometrial carcinoma involvement in the urinary bladder HE: hematoxylin and eosin; CK: cytokeratin

Two years after the initial TUBRT, she underwent a re-TURBT with the aim of pathologically diagnosing the presence or absence of muscle layer invasion. Hematoxylin and eosin (HE) staining revealed invasive proliferation of dysplastic epithelium with glandular-like structures, leading to the diagnosis of invasive urothelial carcinoma with granular differentiation (Figure 3).

Subsequently, the patient was referred to our institution for a definitive therapy for the bladder tumor. However, given the patient's medical history, a differential diagnosis of bladder metastasis of the endometrioid cancer was considered. To reach a definitive diagnosis, additional immunohistochemistry analysis revealed positive staining for cytokeratin (CK) 7, vimentin, and estrogen receptor (ER) and negative staining for CK20 and p63 (Figure 3). Therefore, the final diagnosis was bladder metastasis of endometrioid cancer. After consultation with a gynecologist, RARC with urinary diversion was determined to be the appropriate treatment. 

RARC with bilateral ureterocutaneostomy was performed; however, severe adhesions were present around the ureter due to previous surgeries. The postoperative course was uneventful, and the patient was discharged nine days postoperatively. Pathologic findings by HE staining showed an adherent glandular structure extending from the bladder muscle layer toward the mucosal side. Immunohistochemical findings were similar to those obtained during TURBT. Radiographically, no direct invasion of the primary tumor into the bladder and peritoneal dissemination were observed. Therefore, the patient was diagnosed with bladder metastasis of endometrial cancer. The final diagnosis was bladder metastasis of endometrial adenocarcinoma. After the removal of the bilateral single-J catheters post-discharge, the patient has remained tube-free. No recurrence was observed up to three years after RARC.

## Discussion

Tumors outside the bladder wall often result from direct infiltration of colorectal or uterine cancers, whereas metastases from other organs to the urinary bladder are relatively rare, accounting for approximately 2% of bladder tumors [3]. A comprehensive report on 144 cases of metastatic bladder tumor indicates that malignant melanoma is the most common primary site, occurring in 55 cases, followed by gastric cancer in 34 cases, breast cancer in 16 cases, renal cancer in 14 cases, and other sites in the remaining 25 cases [4]. Since late recurrence of endometrial cancer is uncommon and this case had been recurrence-free for seven years post-initial surgery, primary bladder tumors were initially considered more likely than bladder metastasis of the endometrial cancer.

The initial pathology report from the previous medical facility indicated difficulty in distinguishing between urothelial carcinoma with glandular differentiation and endometrioid adenocarcinoma. The complexity of this diagnosis is due to the histologic similarities between metastatic bladder tumors and tumors differentiated from urothelial carcinoma. Distinguishing between metastatic and primary bladder tumors based solely on clinical and imaging findings is difficult, highlighting the importance of incorporating immunostaining for an accurate pathological diagnosis [5-7]. In this case, in addition to routine HE staining, immunostaining with vimentin, ER, CK7, CK20, and p63 was performed. Based on the high level of staining of vimentin and ER that is commonly associated with endometrioid adenocarcinoma, the lack of staining of p63, a urothelial carcinoma marker, and the presence of CK7 but not CK20, we diagnosed the patient with bladder metastasis from endometrial cancer. Treatment options for metastatic endometrial cancer include chemotherapy, radiation therapy, and hormone therapy for multiple lesions, whereas surgery is preferred for solitary lesions [8]. Complete surgical resection is preferred whenever possible for solitary recurrences because of the mortality rate of approximately 25% in patients with recurrent endometrial cancer [9]. In our case, considering the solitary tumor of the bladder and the potential for curative removal through surgical intervention, RARC was selected. 

Other surgical procedures such as TURBT or partial cystectomy may be considered depending on the depth, location, and size of the metastatic tumor. Previous reports for the treatment of patients with bladder metastases from endometrial cancer have reported chemotherapy alone, TURBT combined with hormonal therapy and radiation therapy, or TURBT combined with chemotherapy and partial cystectomy [2] (Table 1). 

**Table 1:** Cases of bladder metastasis from endometrial cancer PLND: pelvic lymph node dissection; CT: chemotherapy; TURBT: transurethral resection of bladder tumor; HT: hormonal therapy; RARC: robot-assisted radical cystectomy

Author	Age	Initial stage	Initial treatment	Time to metastasis (month)	Treatment	Outcome
Young et al.	68	ⅠA	Radical hysterectomy	16	CT	Local recurrence
Kida et al.	49	ⅣA	Radical hysterectomy+PLND	0	TURBT+HT+RT	No recurrence
Yatsugu et al.	72	ⅠA	Radical hysterectomy+PLND	50	TURBT+CT	N/A
Tokunaga et al.	71	ⅠA	Radical hysterectomy+PLND	72	TURBT+partial cystectomy	N/A
This case	50	Ⅱ	Radical hysterectomy+PLND	84	TURBT+CT+RARC	No recurrence

Because of the small number of cases reported previously and the lack of cases with long-term follow-up, no certain opinion has been obtained regarding the most effective treatment modalities. However, the differentiation between primary and metastatic bladder tumors is very important for determining the therapeutic strategy. Due to the difficulty in diagnosing this disease based on symptoms and/or imaging studies, pathological findings, especially immunohistochemical evaluation, are important to definitively diagnose the bladder metastasis from other organs. To the best of our knowledge, this case represents the first documented instance of successful treatment using RARC for bladder metastasis from endometrial cancer.

## Conclusions

Differentiating between primary and metastatic bladder tumors is essential in determining treatment strategies. Diagnosing solely based on symptoms or imaging tests can be challenging, making pathological diagnosis, especially through immunohistochemistry, crucial for achieving a definitive diagnosis. By making the correct diagnosis, it becomes possible to offer the best treatment to the patients. This case seemed to suggest the importance of considering metastasis to the bladder in patients with a history of other malignant neoplasms, even though the incidence may be less frequent.
